# Anemia among indigenous women in Brazil: findings from the First National Survey of Indigenous People’s Health and Nutrition

**DOI:** 10.1186/s12905-016-0287-5

**Published:** 2016-02-01

**Authors:** Maria Carolina Borges, Romina Buffarini, Ricardo V. Santos, Andrey M. Cardoso, James R. Welch, Luiza Garnelo, Carlos E. A. Coimbra, Bernardo L. Horta

**Affiliations:** 1 Programa de Pós-Graduação em Epidemiologia, Departamento de Medicina Social, Faculdade de Medicina, Universidade Federal de Pelotas, Rua Marechal Deodoro 1160, Pelotas, RS 96001-970 Brazil; 2 Escola Nacional de Saúde Pública, Fundação Oswaldo Cruz, Rua Leopoldo Bulhões 1480, Rio de Janeiro, RJ 21041-210 Brazil; 3 Departamento de Antropologia, Museu Nacional, Universidade Federal do Rio de Janeiro, Quinta da Boa Vista s/n, Rio de Janeiro, RJ 20940-040 Brazil; 4 Centro de Pesquisas Leônidas e Maria Deane, Fundação Oswaldo Cruz, Rua Terezina 476, Manaus, AM 69057-070 Brazil

**Keywords:** Brazil, Indigenous peoples, Health surveys, Nutrition surveys, Health status indicators, Anemia, Maternal health

## Abstract

**Background:**

Anemia is recognized as a major public health problem that disproportionately affects vulnerable populations. Indigenous women of reproductive age in Brazil are thought to be at high risk, but lack of nationwide data limits knowledge about the burden of disease and its main determinants. This study aimed to assess the prevalence of anemia and associated factors in this population using data from The First National Survey of Indigenous People’s Health and Nutrition in Brazil.

**Methods:**

Data were collected from Indigenous women between 15 and 49 years old based on a nationwide sample of villages. The outcomes of interest were hemoglobin levels (g/dL) and anemia (< 12 g/dL for nonpregnant and < 11 g/dL for pregnant women). Multilevel models were used to explore associations with contextual (village) and individual (household/woman) level variables.

**Results:**

Based on data for 6692 Indigenous women, the nationwide mean hemoglobin level was 12.39 g/dL (95 % CI: 12.29–12.50). Anemia prevalence was high (33.0 %; 95 % CI: 30.40–35.61 %) and showed pronounced regional disparities. No village-level characteristics were associated with anemia or hemoglobin levels in the multilevel model. Even after controlling for upper level variables, socioeconomic status, parity, body mass index, and having been treated for malaria were associated with anemia and hemoglobin levels.

**Conclusion:**

The prevalence of anemia in Brazilian Indigenous women was 12 % greater than the national estimates for women of reproductive age. Anemia prevalence and mean hemoglobin levels among Indigenous women appear to be partly explained by some previously recognized risk factors, such as socioeconomic status, body mass index, and malaria; however, part of the variability in these outcomes remains unexplained. Knowledge of health status and its potential determinants is essential to guide public policies aimed at controlling anemia burden in Indigenous communities.

## Background

Anemia is recognized as a major public health problem, affecting over 1.2 billion people worldwide [1]. It has been estimated that more than one third of women of reproductive age suffer from anemia globally, with this burden falling disproportionately on those in low- and middle-income countries [2]. Maternal anemia has well-established short- and long-term consequences for both mother and child, decreasing the chance of survival and increasing the risk of poor fetal growth and complications during pregnancy and the perinatal period [3].

Brazil has undergone major changes in social determinants of health and in the organization of health systems in recent decades [4, 5]. However, Indigenous peoples, one of the most vulnerable groups in the country, still experience high rates of morbidity and mortality due to infectious and parasitic diseases, food insecurity, as well as poor sanitation and housing conditions [6–8].

Previous studies have found that anemia is highly prevalent in Indigenous populations in Brazil, especially among children [9, 10]. There is limited information available on the epidemiology of anemia among adult Indigenous women, despite the vulnerabilities of this group, which often include bearing a first child at an early age and high parity throughout their reproductive years [11–13]. Among the Xavante Indians from Central Brazil, 54.2 % of adult women from 20–40 years of age had anemia [14]. In the Suruí from Southern Amazonia, anemia prevalence rates of 67.3 % and 81.8 % were observed for non-pregnant and pregnant women, respectively, in conjunction with a negative association between the occurrence of anemia and socioeconomic status [15].

The First National Survey of Indigenous People’s Health and Nutrition in Brazil (henceforth, “National Survey”), conducted in 2008–2009, was the first and most comprehensive study to investigate nutritional status based on a nationwide representative sample of Indigenous women (14–49 years of age) and children (<5 years of age) in the country [16]. The present article used National Survey data in order to assess the prevalence of anemia, mean hemoglobin levels and associated factors among Indigenous women.

## Methods

### The National Survey

The National Survey employed a multistage sampling to obtain a representative sample of the country’s official geopolitical regions North, Northeast, Central-West, and South/Southeast (the South and Southeast regions were joined) based on a list of Indigenous villages provided in 2008 by the Brazilian National Health Foundation (Fundação Nacional de Saúde – FUNASA) [16]. The original list contained 3995 villages located in federally recognized Indigenous Reserves, from which 1227 were excluded for being vacated, deactivated, or having less than 31 inhabitants, which was the minimum village size number investigated.

The sample size for each region was calculated using the following parameters: a prevalence of 50 % for all disease outcomes, a relative error of 5 % and a confidence interval of 95 %, according to the methodology proposed by Lemeshow [17]. Sequential Poisson Sampling criteria were used to select villages for inclusion in the study, based on the calculated sample size for each region [18]. The final sample consisted of 123 villages distributed by region as follows: 65 (North), 14 (Central-West), 23 (Northeast), and 21(South/Southeast). In villages with a population of less than 150 eligible individuals, all households were investigated, while, in those with more than 150 eligible individuals, households were selected systematically. The present analysis is based on data collected for women from 14 to 49 years of age.

Of the four questionnaires applied in the National Survey (village, household, woman, child), the data used in this paper derive from the first three. Questions in Portuguese addressed sociodemographic conditions, sanitation, domestic economy, access to health services, and maternal characteristics, among others. Local Indigenous translators (often Indigenous health agents or primary education teachers) were used for interviews with non-Portuguese speaking respondents. For the assessment of hemoglobin, one drop of capillary blood was obtained with one-way lancets fitted to an Accu-Chek lancing device by Roche (Mannheim, Germany) and analyzed using portable hemoglobinometer, model HemoCue Hb 201+ (Ängelholm, Sweden). Body weight was measured to the nearest 100 g with a portable digital scale (Seca model 872, Hamburg, Germany), with participants wearing minimal clothing and barefoot. Standing height was measured with an AlturaExata portable anthropometer (Belo Horizonte, Brazil) and recorded to the nearest 0.1 cm. Previously trained and standardized field researchers carried out anthropometric measurements. A detailed description of the methods used in the National Survey is presented elsewhere [16].

### Study variables

Hemoglobin levels were considered as a continuous (g/dL) as well as a dichotomous variable (anemia: < 12 g/dL for nonpregnant and < 11 g/dL for pregnant women) [1].

Contextual village-level independent variables included access to government social programs related to economic production (Indigenous Initiatives Project – Carteira Indígena), community healthcare (Indigenous Community Healthcare Initiatives Project – Projeto Iniciativas Comunitárias em Saúde Indígena), and food security (Food Acquisition Project – Programa de Aquisição de Alimentos) in the three years prior to the interview.

Household-level independent variables included information on socioeconomic status (household goods index and regular income), living conditions (housing condition and sanitation indices), and food patterns (household consumption/production and seasonal shortages). Regular household income was defined as presence of monthly or annually income from salaries, pensions, or social benefits.

Principal component analysis was used to create the following socioeconomic and living condition variables: (a) household goods index, based on the quantities of durable goods in each household (1^st^ component explained 19 % of variance, eigenvalue: 3.56); (b) housing conditions index, based on type of flooring, walls, roofing, presence of electricity, and fuel used for cooking (1^st^ component explained 48.0 % of variance, eigenvalue: 1.44); and (c) sanitation index, based on primary defecation location, trash disposal destination, primary source of drinking water, and availability of filtered water in the house (1^st^ component explained 56.5 % of variance, eigenvalue: 0.63). Households received scores based on the sum of the contribution of each item multiplied by the quantity of each item before being classified according to tertiles of the combined distribution, considering the four regions combined.

Principal component analysis was also utilized to derive household food consumption and production patterns using 14 food items: rice, corn, manioc, tubers (sweet potatoes and yams), beans, fruits, nuts, vegetables, milk, egg, chicken, beef, game, and fish. Varimax rotation was used to improve component interpretation. The first three components were considered after the analysis of the scree plot, which together explained 44.6 % of the variability. Food items were considered representative of each component if they showed a loading greater than 0.3. Household scores were calculated following the same methodology described above for three subsets of food items: purchased foods index (rice, beans, milk, egg, chicken, and beef purchased within or outside the village); wild foods index (nuts, game, and fish obtained locally by fishing, gathering, and hunting); garden produce index (corn, manioc, tubers, and fruits produced in Indigenous gardens).

Individual-level variables included age group (14–19, 20–34, and 35–49 years), schooling (none, primary, secondary, and tertiary education), body mass index (BMI) (underweight, normal weight, overweight, and obese), parity (number of children ever born), previous treatment for malaria (yes/no), and currently pregnant (yes/no). BMI cutoffs were defined separately for individuals 20 years or more [19] and those 14 to 19 years [20, 21]. For the subset of women with at least one child under 5 years, the following variables about antenatal care during the last pregnancy were included: at least one antenatal care consultation (yes/no), at least 3 tetanus vaccination doses (yes/no), and prescription of iron and folic acid supplements (yes/no).

### Data analysis

Initially, we evaluated the association of each independent variable with blood hemoglobin levels using linear regression and with presence of anemia using logistic regression. Estimates from these bivariate models were corrected for the complex sampling design using Stata survey commands (svy). Independent variables with *p*-values less than 0.20 were included in multivariate analyses. Multilevel models were used considering three levels: region, village characteristics and household and individual characteristics. For each outcome, four models were adjusted: null model, model 1 (village variables), model 2 (village + household variables), and model 3 (village + household + individual variables). For all multilevel models, random effects were considered for the intercept and intraclass correlation coefficients were estimated for region and village levels.

The xtme family commands were used in the multilevel models. Stata software (v. 12.1) was used for all analyses.

### Ethics

The study protocol and collective consent form were approved by the National Ethics Committee (*Comissão Nacional de Ética em Pesquisa* – CONEP, authorization number 256/2008) and the National Indian Foundation (*Fundação Nacional do Índio* – FUNAI). Upon arrival in each village, the research team presented the study objectives and procedures during an open meeting with community leaders and others, preferentially in public, in accordance with each community’s protocols for community decision-making. In addition to describing the objectives and procedures of the study, a Free and Informed Collective Consent form was presented in detail. All questions posed by leaders and community members were answered. If a community granted consent, one or more community leaders publicly signed the consent instrument. During visits to households whose residents were not present when the collective consent form was signed by leaders, the study objectives and procedures were explained and questions answered. Any particular village, household, parent, or guardian was allowed to decline to participate at any moment of fieldwork [16]. Under no circumstances were individuals interviewed or measured without their consent.

## Results

Data from 113 villages were obtained (91.9 % of the original sample). Ten villages were not investigated due to refusal, lack of access, cost, and data loss. Of a total of 5674 Indigenous households originally planned, 6.5 % were not interviewed. The main reasons were residents’ absence at the time of the field team’s visit (5.9 %) and refusal to participate (0.6 %). The number of interviewed women (*n* = 6692) exceeded what was originally planned (*n* = 6605) because the original list of Indigenous village populations were lower than those encountered during data collection. Hemoglobin measurements were taken for 6651 women (99.4 % of the total number of women interviewed).

**Fig. 1:**
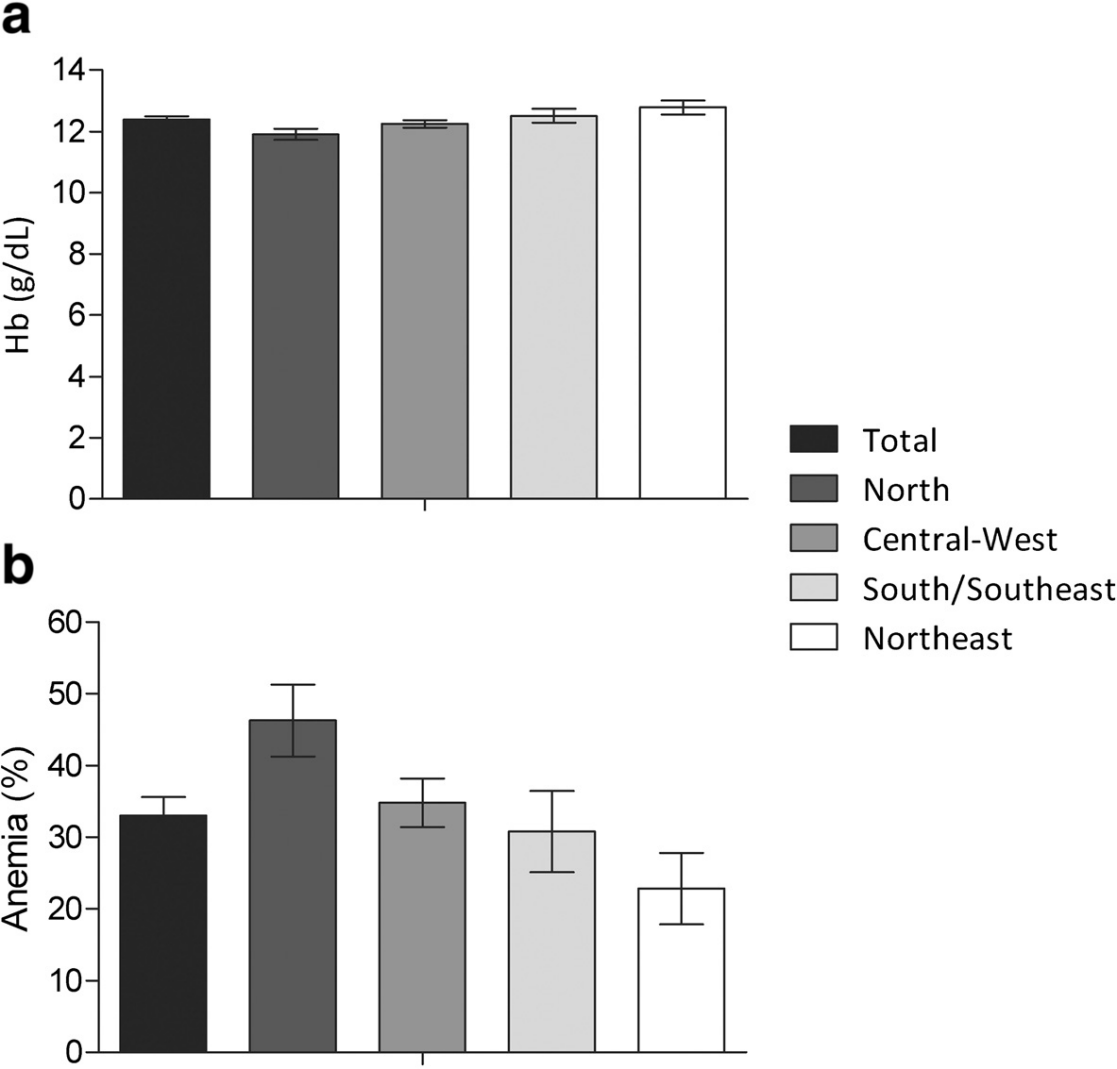
Mean hemoglobin levels (**a**) and anemia prevalence (**b**) with 95 % confidence intervals in Indigenous women according to region. First National Survey of Indigenous People’s Health and Nutrition, Brazil, 2008–2009 (*p*-values < 0.001 for the association between region and hemoglobin levels or anemia prevalence, obtained from linear and logistic regression, respectively)

The overall mean hemoglobin level was 12.39 g/dL (95 % CI: 12.29–12.50), with slight differences observed among regions (Fig. 1a). Nationwide anemia prevalence was high (33.0 %; 95 % CI: 30.40–35.61 %). Regionally, this prevalence in the North (46.3 %; 95 % CI: 41.30–51.26 %) was twice that in the Northeast (22.8 %; 95 % CI: 17.84–27.82 %) (Fig. 1b). The prevalence in the Southeast/South and Central-West regions were, respectively, 30.8 % (95 % CI: 25.15–36.49 %) and 34.8 % (95 % CI: 31.45–38.25 %).

In the bivariate analysis, village access to community healthcare initiatives was associated with higher mean hemoglobin levels. Other village characteristics were not associated with either hemoglobin levels or anemia (Table 1).

**Table 1:** Anemia prevalence and mean hemoglobin levels of Indigenous women according to village characteristics. First National Survey of Indigenous People’s Health and Nutrition, Brazil, 2008–2009

		Anemia		Hemoglobin (g/dL)	
	n^a^	OR (95 % CI)	*p*-value*	β (95 % CI)	*p*-value*
Indigenous Initiatives Project			0.255		0.547
Yes	1729	Reference (1)		Reference (0)	
No	4809	1.16 (0.90; 1.51)		-0.07 (-0.29; 0.15)	
Indigenous Community Healthcare Initiatives Project			0.130		0.044
Yes	2090	Reference (1)		Reference (0)	
No	4534	1.27 (0.93; 1.72)		-0.28 (-0.55; -0.01)	
Food Acquisition Project			0.681		0.653
Yes	1733	Reference (1)		Reference (0)	
No	4905	0.95 (0.73; 1.23)		0.05 (-0.18; 0.28)	
Population size			0.344		0.224
1^st^ tertile	633	Reference (1)		Reference (0)	
2^nd^ tertile	1866	0.72 (0.47; 1.12)		0.33 (-0.04; 0.71)	
3^rd^ tertile	3872	0.86 (0.62; 1.20)		0.16 (-0.14; 0.45)	

*OR* odds ratio, *CI* confidence interval

*Wald Test of heterogeneity

^a^Maximum N for each category, which may vary between variables due to missing data

Higher hemoglobin levels and decreased odds of anemia were consistently related to higher household goods, housing condition, and sanitation indices values. No relationship was observed for regular household income. Food patterns were associated with outcomes, both in terms of quantity (presence of seasonal food shortage) and quality (e.g. the wild foods index was a risk factor, while the purchased foods index was protective against anemia) (Table 2).

**Table 2:** Anemia prevalence and mean hemoglobin levels of Indigenous women according to household characteristics. First National Survey of Indigenous People’s Health and Nutrition. Brazil, 2008–2009

		Anemia Prevalence		Hemoglobin (g/dL)	
	n^a^	OR (95 % CI)	*p*-value*	β (95 % CI)	*p*-value*
Housing condition index			0.001		<0.001
1^st^ tertile	2245	Reference (1)		Reference (0)	
2^nd^ tertile	2160	0.78 (0.62; 0.98)		0.27 (0.12; 0.43)	
3^rd^ tertile	2188	0.63 (0.49; 0.80)		0.43 (0.24; 0.62)	
Sanitation index			0.145		0.012
1^st^ tertile	2995	Reference (1)		Reference (0)	
2^nd^ tertile	1768	0.92 (0.71; 1.20)		-0.02 (-0.22; 0.18)	
3^rd^ tertile	1805	0.79 (0.63; 1.01)		0.25 (0.04; 0.46)	
Purchased foods index			<0.001		<0.001
1^st^ tertile	2360	Reference (1)		Reference (0)	
2^nd^ tertile	2768	0.90 (0.77; 1.04)		0.11 (-0.02; 0.24)	
3^rd^ tertile	1502	0.61 (0.50; 0.75)		0.42 (0.26; 0.57)	
Wild foods index			<0.001		<0.001
1^st^ tertile	2612	Reference (1)		Reference (0)	
2^nd^ tertile	2873	1.48 (1.26; 1.73)		-0.26 (-0.39; -0.13)	
3^rd^ tertile	1145	1.80 (1.37; 2.37)		-0.49 (-0.71; -0.27)	
Garden produced index			<0.001		<0.001
1^st^ tertile	2199	Reference (1)		Reference (0)	
2^nd^ tertile	2185	0.68 (0.58; 0.79)		0.31 (0.18; 0.43)	
3^rd^ tertile	2246	0.94 (0.79; 1.13)		0.09 (-0.05; 0.24)	
Household goods index			<0.001		<0.001
1^st^ tertile	2234	Reference (1)		Reference (0)	
2^nd^ tertile	2191	0.76 (0.65; 0.89)		0.23 (0.11; 0.35)	
3^rd^ tertile	2226	0.58 (0.47; 0.71)		0.46 (0.30; 0.63)	
Regular household income			0.217		0.195
Yes	3134	Reference (1)		Reference (0)	
No	3499	1.08 (0.95; 1.23)		-0.07 (-0.16; 0.03)	
Food shortages			0.026		0.001
Yes	4673	Reference (1)		Reference (0)	
No	1908	0.83 (0.71; 0.98)		0.16 (0.06; 0.27)	

*OR* odds ratio, *CI* confidence interval

*Wald Test of heterogeneity

^a^Maximum N for each category, which may vary between variables due to missing data

With regard to individual characteristics, having received malaria treatment was a strong predictor of anemia and hemoglobin levels, while parity was only associated modestly with hemoglobin levels. Overweight and obese women had higher hemoglobin levels and lower anemia prevalence when compared to normal weight women (Table 3). For the subsample of women with children < 5 years, attending at least one prenatal care consultation was associated with higher hemoglobin levels (0.29 g/dL; 95 % CI: 0.02–0.57), while having received the anti-tetanus vaccine was nominally associated with higher odds of anemia (OR 1.20; 95 % CI: 1.00–1.43) in the bivariate analysis. The other antenatal care variables (prescription of iron or folic acid supplement during the most recent pregnancy) were not associated with hemoglobin levels or anemia (data not shown in table).

**Table 3:** Anemia prevalence and mean hemoglobin levels of Indigenous women according to individual characteristics. First National Survey of Indigenous People’s Health and Nutrition, Brazil, 2008–2009

		Anemia Prevalence		Hemoglobin (g/dL)	
	n^a^	OR (95 % CI)	*p*-value*	β (95 % CI)	*p*-value*
Age group (years)			0.250		0.021
14–19	1691	Reference (1)		Reference (0)	
20–34	3468	0.95 (0.82; 1.09)		0.05 (-0.07; 0.16)	
≥ 35	1492	0.88 (0.75; 1.03)		0.16 (0.03; 0.29)	
Schooling (years)			0.302		0.059
0	998	Reference (1)		Reference (0)	
1–4	2597	0.97 (0.81; 1.16)		0.01 (-0.16; 0.18)	
5–9	1811	0.89 (0.74; 1.08)		0.13 (-0.06; 0.32)	
≥ 10	1197	0.80 (0.61; 1.05)		0.19 (-0.05; 0.42)	
BMI			<0.001		<0.001
Underweight	183	0.78 (0.51; 1.21)		0.14 (-0.15; 0.43)	
Normal weight	3618	Reference (1)		Reference (0)	
Overweight	1977	0.78 (0.67; 0.92)		0.14 (0.02; 0.26)	
Obese	845	0.62 (0.50; 0.77)		0.39 (0.23; 0.55)	
Parity	6621	1.03 (1.00; 1.07)	0.060	-0.03 (-0.06; 0.00)	0.018
Malaria treatment			<0.001		<0.001
Yes	568	Reference (1)		Reference (0)	
No	6051	0.43 (0.29; 0.62)		0.62 (0.29; 0.96)	
Current pregnancy			0.277		<0.001
Yes	646	Reference (1)		Reference (0)	
No	5720	0.89 (0.73; 1.10)		1.07 (0.93; 1.20)	

*OR* odds ratio, *CI* confidence interval, *BMI* Body mass index

*Wald Test of heterogeneity

^a^Maximum N for each category, which may vary between variables due to missing data

The intraclass correlation coefficient (ICC) was 0.12–0.14 for village and 0.04–0.05 for region level in the null multilevel model. ICC decreased after inclusion of the study variables (ICC_village_ 0.09–0.11; ICC_region_ 0.02). None of the village-level variables were associated with anemia or hemoglobin levels after controlling for region in the multilevel model (Tables 4 and 5). Likewise, household sanitation condition, seasonal food shortage, and food pattern indices lost significance after controlling for region and village-level variables. Moderate and higher housing condition index scores (2^nd^ tertile), household goods index (2^nd^ and 3^rd^ tertiles), and garden produced index (2^nd^ tertile) were associated with higher hemoglobin concentration values. Only the household goods index and wild foods index were associated with anemia. Higher parity was associated with lower hemoglobin values and presence of anemia; current pregnancy was associated with lower hemoglobin levels only. BMI and having received treatment for malaria were associated with both outcomes (Tables 4 and 5). In the subsample of women with children < 5 years, antenatal care variables (receiving at least one prenatal care consultation and anti-tetanus immunization) were no longer associated with the outcomes in the multivariate model.

**Table 4:** Mean hemoglobin levels according to village, household and individual level variables. First National Survey of Indigenous People’s Health and Nutrition. Brazil, 2008–2009. Geopolitical region was included as a control variable at all levels

	Model 1		Model 2		Model 3	
	β	(95 % CI)	β	95 % CI	β	95 % CI
*Level 1: Village*						
Absence of Indigenous Initiatives Project	-0.11	(-0.35; 0.14)	-0.08	(-0.32; 0.17)	-0.11	(-0.35; 0.13)
Absence of Indigenous Community Healthcare Initiatives Project	-0.06	(-0.29; 0.17)	-0.05	(-0.28; 0.18)	-0.09	(-0.31; 0.14)
Absence of Food Acquisition Project	0.07	(-0.15; 0.29)	0.06	(-0.16; 0.29)	0.06	(-0.16; 0.28)
*Level 2: Household characteristics*						
Housing condition index						
1^st^ tertile			Reference (0)		Reference (0)	
2^nd^ tertile			0.10	(0.00; 0.20)	0.11	(0.00; 0.21)
3^rd^ tertile			0.06	(-0.07; 0.18)	0.06	(-0.06; 0.19)
Sanitation index						
1^st^ tertile			Reference (0)		Reference (0)	
2^nd^ tertile			-0.01	(-0.13; 0.09)	-0.04	(-0.15; 0.07)
3^rd^ tertile			-0.02	(-0.14; 0.11)	-0.06	(-0.18; 0.07)
Household goods index						
1^st^ tertile			Reference (0)		Reference (0)	
2^nd^ tertile			0.07	(-0.02; 0.17)	0.08	(0.01; 0.18)
3^rd^ tertile			0.19	(0.08; 0.30)	0.15	(0.03; 0.26)
Absence of regular income			-0.05	(-0.12; 0.03)	-0.00	(-0.07; 0.07)
Absence of food shortages			0.05	(-0.02; 0.13)	0.05	(-0.03; 0.13)
Purchased foods index						
1^st^ tertile			Reference (0)		Reference (0)	
2^nd^ tertile			0.01	(-0.09; 0.12)	-0.01	(-0.12; 0.09)
3^rd^ tertile			0.05	(-0.07; 0.17)	0.05	(-0.07; 0.17)
Wild food index						
1^st^ tertile			Reference (0)		Reference (0)	
2^nd^ tertile			-0.05	(-0.16; 0.07)	-0.00	(-0.12; 0.10)
3^rd^ tertile			-0.07	(-0.21; 0.07)	-0.05	(-0.19; 0.09)
Garden produce index						
1^st^ tertile			Reference (0)		Reference (0)	
2^nd^ tertile			0.12	(0.03; 0.21)	0.12	(0.03; 0.22)
3^rd^ tertile			-0.04	(-0.15; 0.07)	-0.05	(-0.16; 0.06)
*Level 3: Individual characteristics*						
Age group (years)						
14–19					Reference (0)	
20–34					0.07	(-0.03; 0.17)
35–49					0.11	(-0.03; 0.25)
Schooling (years)						
0					Reference (0)	
1–4					-0.07	(-0.18; 0.04)
5–9					-0.03	(-0.16; 0.10)
≥ 10					-0.07	(-0.22; 0.07)
BMI						
Normal					Reference (0)	
Underweight					-0.09	(-0.29; 0.11)
Overweight					0.15	(0.08; 0.23)
Obese					0.25	(0.14; 0.36)
Parity					-0.06	(-0.09; -0.02)
Absence of malaria treatment					0.23	(0.09; 0.38)
No current pregnancy					0.98	(0.87; 1.09)
Intercept	12.5	(12.0; 13.0)	12.3	(11.8; 12.9)	11.3	(10.8; 11.9)
Deviance (-2 loglikelihood)	22513		21832		20361	
ICC village	0.13		0.12		0.11	
ICC region	0.04		0.03		0.03	

*β* regression coefficient, *CI* confidence interval, *BMI* body mass index

Null model (*n* = 6651)

Model 1: village (Level 1) (*n* = 6515)

Model 2: village (Level 1) + household (Level 2) (*n* = 6332)

Model 3: village (Level 1) + household (Level 2) + individual (Level 3) (*n* = 5998)

**Table 5:** Anemia prevalence according to village, household and individual level variables. First National Survey of Indigenous People’s Health and Nutrition. Brazil, 2008–2009. Geopolitical region was included as a control variable at all levels

	Model 1		Model 2		Model 3	
	OR	(95 % CI)	OR	(95 % CI)	OR	(95 % CI)
*Level 1: Village*						
Indigenous Community Healthcare Initiatives Project	1.03	(0.76; 1.38)	1.01	(0.75; 1.35)	1.03	(0.76; 1.38)
*Level 2: Household characteristics*						
Housing condition index						
1^st^ tertile			Reference (1)		Reference (1)	
2^nd^ tertile			0.95	(0.80; 1.12)	0.95	(0.80; 1.12)
3^rd^ tertile			0.93	(0.76; 1.14)	0.93	(0.75; 1.13)
Sanitation index						
1^st^ tertile			Reference (1)		Reference (1)	
2^nd^ tertile			0.99	(0.83; 1.18)	1.01	(0.85; 1.21)
3^rd^ tertile			1.17	(0.96; 1.43)	1.19	(0.97; 1.46)
Household goods index						
1^st^ tertile			Reference (1)		Reference (1)	
2^nd^ tertile			0.87	(0.76; 1.01)	0.88	(0.76; 1.01)
3^rd^ tertile			0.71	(0.60; 0.84)	0.73	(0.61; 0.87)
Absence of food shortages			0.93	(0.82; 1.06)	0.94	(0.82; 1.07)
Purchased foods index						
1^st^ tertile			Reference (1)		Reference (1)	
2^nd^ tertile			0.94	(0.80; 1.11)	0.95	(0.81; 1.13)
3^rd^ tertile			0.95	(0.78; 1.16)	0.95	(0.78; 1.16)
Wild food index						
1^st^ tertile			Reference (1)		Reference (1)	
2^nd^ tertile			1.25	(1.05; 1.50)	1.24	(1.04; 1.49)
3^rd^ tertile			1.15	(0.92; 1.43)	1.17	(0.94; 1.46)
Garden produce index						
1^st^ tertile			Reference (1)		Reference (1)	
2^nd^ tertile			0.88	(0.75; 1.02)	0.88	(0.75; 1.02)
3^rd^ tertile			1.04	(0.87; 1.24)	1.04	(0.87; 1.25)
*Level 3: Individual characteristics*						
Age group (years)						
14–19					Reference (1)	
20–34					0.98	(0.83; 1.16)
35–49					0.93	(0.74; 1.16)
Schooling (years)						
0					Reference (1)	
1–4					1.07	(0.89; 1.27)
5–9					1.06	(0.86; 1.30)
≥ 10					1.06	(0.83; 1.35)
BMI						
Normal					Reference (1)	
Underweight					1.00	(0.71; 1.40)
Overweight					0.80	(0.69; 0.90)
Obese					0.73	(0.59; 0.87)
Parity					1.06	(1.00; 1.11)
Absence of malaria treatment					0.66	(0.53; 0.82)
Deviance (-2 loglikelihood)	8192		7909		7748	
ICC village	0.11		0.10		0.09	
ICC region	0.04		0.03		0.02	

*OR*: odds ratio, *CI*: confidence interval, *BMI*: body mass index

Null model (*n* = 6651)

Model 1: village (Level 1) (*n* = 6624)

Model 2: village (Level 1) + household (Level 2) (*n* = 6443)

Model 3: village (Level 1) + household (Level 2) + individual (Level 3) (*n* = 6351)

## Discussion

The results of the National Survey indicate that approximately one third of Indigenous women in Brazil are anemic. This value is just slightly above that derived from the most recent national prevalence estimate for anemia in non-pregnant Brazilian adult women 15–49 years of age, which was 29.4 % (29.7 % in urban and 27.9 % in rural areas) [22]. Notwithstanding, while the findings from the National Survey and the 2006 National Demography and Health Survey [22] point to comparable prevalence rates, there are major regional differences, with Indigenous women presenting much higher prevalence of anemia in the North and Central-West regions (46.3 % vs. 19.3 % and 34.8 % vs. 20.1 %, respectively). Whereas prevalence rates of anemia in Indigenous women and Brazilian women nationally are more similar for the Southeast and South regions, Indigenous women from the Northeast region presented a much lower rate than Brazilian women nationally (22.8 % vs. 39.1 %, respectively). This latter finding is unexpected since the overall socioeconomic and health conditions of Indigenous populations tend to be worse in comparison to the general Brazilian population [16, 23, 24].

The contrasting regional trends observed in the prevalence of anemia among Indigenous women as compared to the general population possibly derive from a complex interplay of socioeconomic, political, and environmental factors [16]. Previous analyses from the National Survey have shown that overall socioeconomic conditions and sanitation infrastructure tend to be better in the Northeast than other regions in Brazil [16]. In addition, Indigenous women in the Northeast presented much higher frequencies of prenatal consultations. For instance, whereas only 33.4 % of Indigenous women in the North had at least one prenatal consultation, this proportion was 58.6 % in the Northeast, where 48.3 % of Indigenous women attained a minimum of six consultations during their most recent pregnancy [16]. In fact, half of the variance of anemia prevalence attributed to the regional level was explained after adjusting for socioeconomic, reproductive, and antenatal care indicators.

In order to achieve a broader understanding of potential important factors associated with anemia among Indigenous women, this study addressed both contextual and individual variables. Concerning village characteristics, none of the study variables were associated with anemia or hemoglobin levels in the multivariate model. In relation to household variables, there was a dose-response relationship between the possession of durable household goods and the studied outcomes. Women with higher parity were also more likely to show lower hemoglobin levels. Given a context of high fertility, Indigenous women experience various conditions that might result in anemia, including multiple pregnancies separated by short birth intervals, high prevalence of endemic parasitic infections, food insecurity, and poor prenatal care [25–27].

It is well established that anemia in adult women is closely associated with socioeconomic conditions, including years of schooling. For instance, in Brazil, the prevalence of anemia in adult women decreased with years of schooling. While the prevalence of anemia in women with 0–4 years of schooling was 33.2 %, it dropped to 27.8 % among women with ≥ 9 years of attendance [28]. In this study we observed that neither levels of hemoglobin nor presence of anemia were associated with schooling. It is unclear why no association was found but it might be that the inclusion of other socioeconomic variables, in particular the household goods index, might have obfuscated the influence of years of schooling.

Despite the acknowledged importance of nutrition on hemoglobin levels, there was no consistent evidence of an association between anemia and reported food scarcity or household food consumption and production patterns. However, food security issues are critical among many Indigenous people worldwide and should not be underestimated [29]. In Brazil, Indigenous families that migrate to urban areas often experience major changes in their dietary patterns. In rural areas, Indigenous communities often face strong pressure from the expansion of agriculture, mining, and extractive (e.g. timber) industries or live in reservations located in environmentally degraded regions [16]. Over 90 % of Brazilian Indigenous communities reported some food scarcity during the year and more than 50 % reported such shortages lasted longer than four months [30]. The current economic scenario in Brazil also has affected the subsistence and dietary practices of Indigenous people, either through agricultural intensification and adoption of extractive production or paid work [16]. Thus, while there is broad evidence of food insecurity in Indigenous communities throughout Brazil [31, 32], it is possible that the National Survey questionnaire did not have sufficient sensitivity to capture its effects at the individual level. For instance, whereas the present study collected data regarding whether the household experiences seasonally food shortage, the nutritional and socioeconomic determinants of anemia at the individual level might be influenced by factors operating on a broader scale of time.

In contrast with the lack of association between food patterns and anemia, excess weight (i.e., overweight and obesity) showed a protective effect. The relationship between weight and anemia is complex [33]. On the one hand, overweight/obese women might have a greater chance of meeting specific nutrient requirements under food insecure settings due to higher food intake. On the other hand, adiposity is related to chronic inflammation and decreased iron absorption [34, 35], which might increase susceptibility to anemia. Population-based studies carried out in low- and middle-income countries have yielded mixed findings, with evidence of both positive [33, 34] and negative [33, 36, 37] associations between weight and anemia.

Having received treatment for malaria, a proxy for malaria infection, was strongly and consistently associated with hemoglobin levels and anemia. Infection with *Plasmodium* species has a well-known impact on anemia, due to targeting of red blood cells [38, 39]. Over half of the Indigenous population in Brazil lives in areas of moderate to high risk of malaria transmission [40–42]. Women may face a higher risk of contracting malaria and, during pregnancy, malaria can cause miscarriage, still birth, or delivery of low-birth-weight infants [43]. This is particularly relevant considering that the total fertility rate of Indigenous women in Brazil is much higher than that of the general population, reaching averages close to 4 children per woman [12, 44]. As the results of this study have shown, higher woman’s parity is associated with lower hemoglobin concentration levels and increased presence of anemia. In areas of *Plasmodium falciparum* transmission, immune suppression induced by anemia favors the evolution of severe clinical malaria, often leading to death.

A recent review of the epidemiology of anemia in Indigenous peoples across the world has indicated iron deficiency, malaria, and helminth infections as the leading causes [45]. Therefore, public health initiatives aiming at reducing the burden of anemia in Indigenous communities should not be limited to the nutritional dimension. Several interventions have proved effective against these types of anemia, such as iron supplementation and food fortification programs, use of insecticide-treated bed-nets for reducing malaria transmission rates, and improved housing and sanitation to reduce both malaria and helminth morbidity [46]. Although these interventions have been implemented as public health initiatives for the general Brazilian population, they are less present in socially marginalized segments, as the Indigenous communities [16].

Some limitations to this study should be taken into account when interpreting its results. The cross-sectional nature of the research design does not allow for the establishment of causal relationships. Moreover, the use of household rather than individual food consumption data precludes dietary analysis of bioavailable iron sources, which is important for understanding the epidemiology of anemia in the study population. Concerning analytical procedures employed in this paper, several study variables, especially social indicators, were chosen based on evidence of anemia determinants in non-Indigenous societies and therefore may not be similarly relevant for this Indigenous population.

## Conclusion

This study reports for the first time on the prevalence of anemia and associated factors among Indigenous women of childbearing age in Brazil based on a nationwide representative sample. Anemia prevalence was slightly higher among Indigenous women than has been documented for women in the general Brazilian population, suggesting they are somewhat disadvantaged in terms of nutritional and overall health. This study also reveals a scenario of marked disparities in anemia prevalence between regions, with nearly half of Indigenous women in northern Brazil being affected. The occurrence of low hemoglobin levels and anemia among Indigenous women are associated with well-known factors, such as socioeconomic status, food patterns, nutritional status, parity, and exposure to malaria. However, part of the variability in these outcomes remains unexplained, which might derive from specific ecological and socioeconomic conditions pertaining to this ethnically differentiated population. Further research on risk factors and potential determinants of anemia among Indigenous communities is essential to guide public policies and health-nutritional interventions aimed at controlling the burden of anemia in these populations.

## Abbreviations

BMI: body mass index
CI: confidence interval
CONEP: Comissão Nacional de Ética em Pesquisa
FUNAI: Fundação Nacional do Índio
FUNASA: Fundação Nacional de Saúde
